# “Prefrontal” Neuronal Foundations of Visual Asymmetries in Pigeons

**DOI:** 10.3389/fphys.2022.882597

**Published:** 2022-05-02

**Authors:** Qian Xiao, Onur Güntürkün

**Affiliations:** ^1^ Department of Biopsychology, Institute of Cognitive Neuroscience, Faculty of Psychology, Ruhr University Bochum, Bochum, Germany; ^2^ Laboratory of Interdisciplinary Research, Institute of Biophysics, Chinese Academy of Sciences, Beijing, China

**Keywords:** birds, tectofugal system, lateralization, single unit recording, nidopallium caudolaterale

## Abstract

This study was conducted in order to reveal the possibly lateralized processes in the avian nidopallium caudolaterale (NCL), a functional analogue to the mammalian prefrontal cortex, during a color discrimination task. Pigeons are known to be visually lateralized with a superiority of the left hemisphere/right eye for visual feature discriminations. While animals were working on a color discrimination task, we recorded single visuomotor neurons in left and right NCL. As expected, pigeons learned faster and responded more quickly when seeing the stimuli with their right eyes. Our electrophysiological recordings discovered several neuronal properties of NCL neurons that possibly contributed to this behavioral asymmetry. We found that the speed of stimulus encoding was identical between left and right NCL but action generation was different. Here, most left hemispheric NCL neurons reached their peak activities shortly before response execution. In contrast, the majority of right hemispheric neurons lagged behind and came too late to control the response. Thus, the left NCL dominated the animals’ behavior not by a higher efficacy of encoding, but by being faster in monopolizing the operant response. A further asymmetry concerned the hemisphere-specific integration of input from the contra- and ipsilateral eye. The left NCL was able to integrate and process visual input from the ipsilateral eye to a higher degree and thus achieved a more bilateral representation of two visual fields. We combine these novel findings with those from previous publications to come up with a working hypothesis that could explain how hemispheric asymmetries for visual feature discrimination in birds are realized by a sequential buildup of lateralized neuronal response properties in the avian forebrain.

## Introduction

Asymmetries of brain and behavior are not only widespread among vertebrates, but also extend to bilaterians and thus presumably to the majority of animals (Vallortigara and Rogers, 2005; Ocklenburg et al., 2013; Güntürkün et al., 2020). This ubiquity of non-human animals with cerebral asymmetries provides a great opportunity to develop animal models in order to reveal the mechanisms with which genetic (Norris, 2012; Brandler and Paracchini, 2014), neural (Moorman et al., 2012; Chou et al., 2016; Washington et al., 2021), and cognitive systems (Regolin et al., 2005; Della Chiesa et al., 2006; Yamazaki et al., 2007) govern lateralized behavior. Avian models like chicks and pigeons proved especially suitable to discern the neural foundations of such asymmetries. Both adult pigeons and chicken hatchlings show left-right differences of behavior in various visual tasks that involve discrimination of object details (Güntürkün, 1985; Rogers, 2014), spatial locations (Prior et al., 2004; Tommasi et al., 2012), or social companions (Daisley et al., 2009). It is relevant to emphasize that both the left and right hemisphere of birds provide specific contributions to visual behavior. While pigeons and chicks reach higher accuracy and speed during pattern or color discrimination tasks when using their left hemisphere/right eye (Vallortigara, 1989; Rogers, 2014; Xiao and Güntürkün, 2018), the right hemisphere/left eye excels when social or spatial stimuli are to be distinguished (Rosa-Salva et al., 2012; Tommasi et al., 2012). Thus, avian brain asymmetries are not about an overall hemispheric dominance, but implies the existence of task- and hemisphere-specific circuits.

**FIGURE 1:**
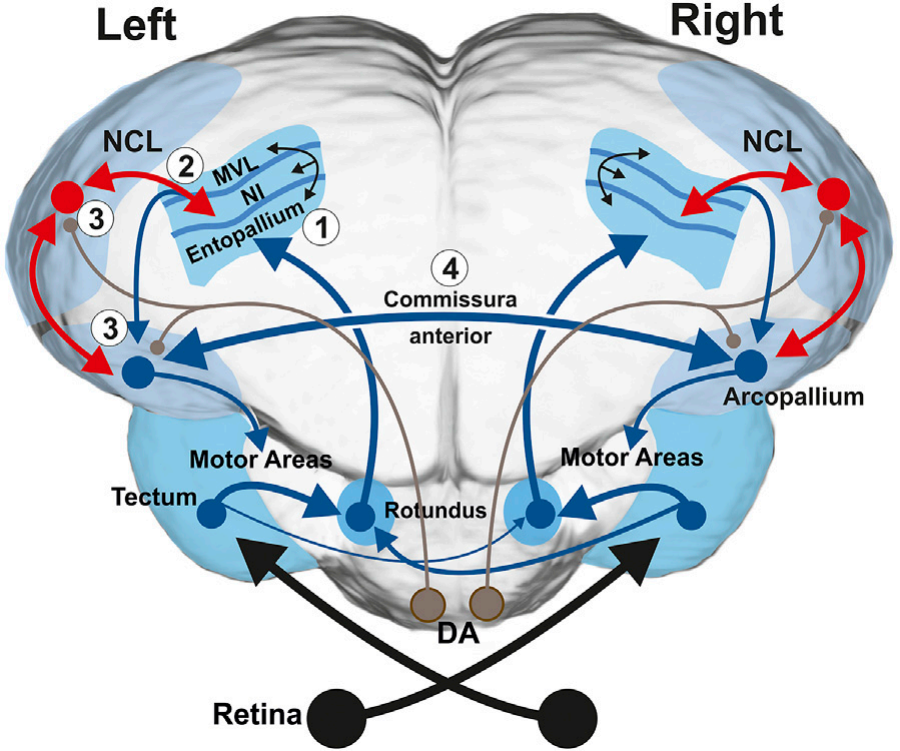
Schematic frontal depiction of the tectofugal visual system along with the anterior commissure, the projection of the tectofugal system to the prefrontal-like nidopallium caudolaterale (NCL) and the projection of NCL onto the motor arcopallium (A). The subsystems that play key roles for the present study are depicted in red. The tectorotundal axons have a larger number of fibers that cross from right to left, thereby constituting a more bilateral representation of visual information in the left hemisphere. In the current study, we recorded the activity patterns of single NCL neurons. The NCL receives input from the tectofugal system via the nidopallium intermediale (NI) and feedback projections from the arcopallium. In addition, dopaminergic (DA) brainstem projections (depicted in brown color) can modify visuo-associative and visuo-motor connections in experience dependent manner. Numbers 1–4 refer to the four steps of the working hypothesis on lateralized visual discrimination learning and task execution at pallial level in birds. Further abbreviation: mesopallium ventrolaterale (MVL).

These asymmetries can be traced down to underlying neural networks. In birds, visual information is processed in two parallel ascending pathways, the thalamofugal and the tectofugal system (Mouritsen et al., 2016). The thalamofugal system transfers input from the retina *via* the contralateral n. geniculatus lateralis, pars dorsalis (GLd) bilaterally to the visual hyperpallium (Güntürkün and Karten, 1991). In chicks, this system is asymmetrically organized with more bilateral inputs to the right hyperpallium (Rogers, 2018) that consequently displays lateralized activity patterns (Costalunga et al., 2021). The second ascending visual pathway is the tectofugal system that projects *via* the contralateral midbrain tectum opticum and the thalamic n. rotundus (Rt) to the entopallium. In pigeons, this pathway evinces various anatomical left-right differences (Güntürkün et al., 2020). Most of tectum axons ascend to the ipsilateral Rt, while a smaller contingent crosses the midline and reaches the contralateral Rt (Figure 1). These contralaterally projecting fibres are more numerous from the right tectum to the left Rt than vice versa (Güntürkün et al., 1998; Letzner et al., 2020). Therefore, a larger bilateral input arrives through the left Rt into the left entopallium (Güntürkün et al., 1998; Güntürkün and Hahmann, 1999; Skiba et al., 2002; Letzner et al., 2020). Verhaal et al. (2012) recorded multi-unit responses from right and left entopallium while pigeons were discriminating colors. Similar to the observations of Colombo et al. (2001), entopallial neurons started to respond with an initial phasic burst when processing the rewarded stimulus. This initial activity peak, however, was only present in left entopallial neurons, making an initial recruitment of a larger population of left entopallial neurons. Such an abrupt unilateral avalanche of entopallial activity could activate a larger number of downstream left-hemispheric associative and motor structures of the left hemisphere, thereby resulting in visually controlled behavior that is mostly governed by the left hemisphere.

The entopallium is part of a cortex-like avian sensory pallium that is constituted by radially and tangentially organized neurites that create an orthogonally organized fiber pattern (Stacho et al., 2020). Local communities of neurons that are embedded in this structure create iteratively repeated columnar canonical circuits (Güntürkün et al., 2021). These circuits are tangentially intersected by axons that cross-connect visual tectofugal columns with the (pre)motor arcopallium and the prefrontal-like nidopallium caudolaterale (NCL) (Figure 1).

The arcopallium projects to various brainstem areas to control visually guided behavior (Wild et al., 1985; Davies et al., 1997). However, the arcopallium also gives rise to the anterior commissure that is the main source for interhemispheric crosstalk in birds (Letzner et al., 2016). Xiao and Güntürkün (2018), Xiao and Güntürkün (2021) recorded from single arcopallial neurons while the pigeons were discriminating colors and discovered two key mechanisms. First, the neurons of the left arcopallium were faster in triggering the conditioned response, thereby providing the left hemisphere a significant time-advantage that translates into a hemisphere-specific control of behavior during visual object discriminations (Xiao and Güntürkün, 2018). Second, visual information was asymmetrically exchanged *via* the commissure with the left arcopallium providing the right side more information about ipsilateral stimuli than vice versa (Xiao and Güntürkün, 2021).

The second major target of tectofugal columns is the NCL (Stacho et al., 2020) (Figure 1). The NCL receives massive dopaminergic input (von Eugen et al., 2020) and constitutes a large avian pallial hub that is situated between all ascending sensory and descending motor systems (Kröner and Güntürkün, 1999). Based on this connectivity pattern, the neurochemical architecture (Herold et al., 2011) and prefrontal-like executive functions (Güntürkün, 1997a; Hartmann and Güntürkün, 1998; Diekamp et al., 2002a), it is likely that the NCL is a functional avian analogue to the mammalian prefrontal cortex (Güntürkün, 2012). This analogy was importantly substantiated by single unit recordings in pigeons and crows. The avian NCL neurons showed identical neuronal properties as monkey prefrontal neurons during working memory performance (Diekamp et al., 2002b; Johnston et al., 2017; Hahn et al., 2021), stimulus value judging (Kalenscher et al., 2005; Dykes et al., 2018), serial order behavior (Johnston et al., 2020), decision making (Lengersdorf et al., 2014; Veit et al., 2015), prediction error signaling (Packheiser et al., 2021), abstract rules (Veit and Nieder, 2013), as well as coding for numerical information (Nieder, 2017), and conscious experiences (Nieder et al., 2020). The bulk of these evidences indicate that mammals and birds evolved strikingly similar “prefrontal” pallial areas in convergent manner (Güntürkün et al., 2021).

Taken together, the avian NCL is the key area for storing, gating and weighing incoming sensory formation and subsequent planning of appropriate actions. Since visual asymmetries in birds affect perceptual, cognitive, and action-related tasks, it is likely that NCL neurons might show left-right differences during visual discrimination. We therefore set out to analyze single NCL neurons during a color discrimination task. The current paper derives from a comprehensive study of which the arcopallial results have been published (Xiao and Güntürkün, 2018; Xiao and Güntürkün, 2021). The data from NCL were up to now not analyzed and reported.

## Methods

### Bird Training

Six adult homing pigeons (*Columba livia*) from local breeders and of unknown sex were employed and maintained on a 12 h day/night cycle. Food was available at all times. On non-training and non-test days, the birds were allowed to drink as much as they wanted. The day before training or testing, the water bowl in the cage was removed in the afternoon to motivate the animals to receive a water reward during the experiment in the next day. On average, the animals received approximately 4 ml of water during a single session. After training or testing, the birds were allowed to drink ad libitum in the cages for several hours. The weight and health of the animals were monitored on a daily basis. All procedures were in accordance with the National Institutes of Health Guidelines for the Care and Use of Laboratory Animals and were approved by a state committee (North Rhine-Westphalia, Germany).

We used a procedure that was first introduced by Mallin and Delius (1983). Before the training started, a metal head-fixation pedestal (1 × 0.7 × 0.4 cm, 1.6 g) was glued onto the animals’ skull with dental cement to prevent head movements. Isoflurane (∼2% by volume in O_2_, Medical Developments International) was used for anesthesia during surgery. Body temperature was maintained at 40°C by an electric warming pad. A recording trough of 5 × 5 mm was built with dental acrylic on the skull to access the target area of each hemisphere. After surgery, animals were allowed to recover for at least 7 days. The experiments were run in a dark room. The color stimuli were provided by light-emitting diodes (LED) located at each side with a distance of 5 cm away from each eye. LEDs were inserted into a tube with a diameter of 11 mm that pointed closely towards one eye to avoid the diffusion of another eye. This arrangement made sure that the light stimulation was limited and only reached one eye. LEDs were controlled by a custom software written in Matlab (Mathworks, R2009a, United States). Pigeons were trained to discriminate four colors (blue, green, yellow and red) each with the luminance of 0.6 cd/m^2^. The colors were 2 × 2 paired and each pair of colors (Go- and NoGo-stimulus) was exclusively learned by one eye/hemisphere. Color pairs were balanced between animals. Using the custom-made water container (1 × 0.6 × 0.4 cm) and the beak monitoring system, each beak movement (mandibulation) was detected by an infrared light barrier (Zweers, 1982) and synchronically recorded with task events including the onset and offset of stimulus and reward delivery. The tip of the beak was placed in the middle of the water container. The water pump needed ∼0.5 s to fill or empty the container. After the water container was full, water remained for 0.5 s.

**FIGURE 2:**
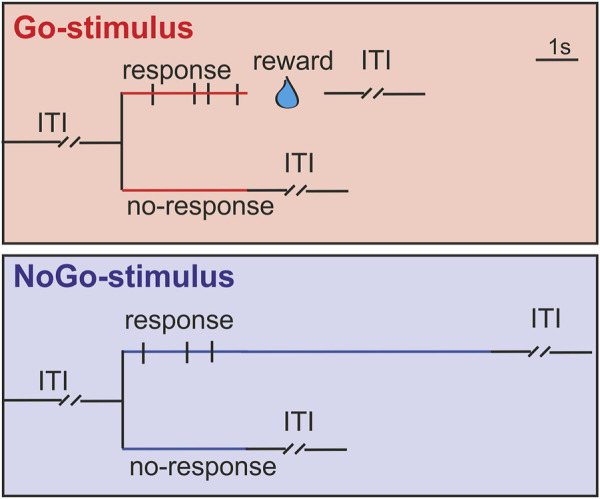
Schematic drawing of the training paradigm. Stimuli were presented to each eye in pseudorandomly interleaved trials with 15 s inter-trial intervals (ITI). Animals were trained to either respond to Go-stimuli (red line) or withhold responses to NoGo-stimuli (blue line) during a 3 s response period after the stimulus onset. Correct responses in Go-trials were rewarded with water at the end of response period and the stimuli were switched off during the reward time. Correct responses (no jaw movements) to NoGo-stimuli were not rewarded, whereas jaw movements during the 3 s response period prolonged stimulus presentation time from 3 s to 9 s. Black vertical bars during the 3 s response period indicate jaw movements of animals (mandibulations).

During experiments, we characterized visual responses of NCL neurons of both hemispheres. A training session consisted of 80 trials with 20 trials for each color. Only one eye was stimulated in each trial. The stimuli were presented in pseudorandomly interleaved trials with 15 s inter-trial intervals (ITIs). Once an LED that pointed to one eye was switched on (stimulus onset), the animals had a 3 s response period to either answer to the Go-stimulus or withhold responses to the NoGo-stimulus. Correct responses in Go-trials were rewarded at the end of the response period while the stimulus was switched off during reward. Correct NoGo responses (“rejections”) were not rewarded, but mandibulations during NoGo trials (“false alarms”) prolonged stimulus presentation time from 3 s to 9 s (Figure 2). After the signal for reward delivery was given, the pump needed 0.33 s to fill half of the water-container and 0.5 s to fill it completely. When the animals’ correct responses to Go- and NoGo-stimuli for each eye both reached 85% on three continuous days, electrophysiological recordings started. The animals’ behavioral responses detected by laser detector and task events were recorded synchronically.

### Extracellular Recording

Before recording, a small craniotomy was made at the location of the targeted area under isoflurane anesthesia as described above. Animals were allowed to recover for 1 week and were retrained again until reaching criteria. At the end of each recording day, the recording trough was filled with dental silicone (0.1%, Sigma). There were 2–4 training days between two continuous recording days for each animal.

Neuronal responses from NCL (A: 5.0–7.0; L: 6.0–8.0; D: 1.0–3.0) (Karten and Hodos, 1967; Kröner and Güntürkün, 1999) of two hemispheres were recorded (Thomas recording, 1–2 MΩ, 7-channels Eckhorn System, Germany) while animals were engaged in discriminating colors. Spikes were amplified (×1,000–2,500), filtered (500–5,000 Hz, single-unit activity filter/amplifier system, Thomas Recording, Germany) and displayed on the oscilloscope. Signals were continuously acquired at 20.8 kHz on a 16-channels Spike2 system (CED, Micro1401-mk2, Cambridge Electronic Design Ltd, United Kingdom). Task events and beak movements were digitized at a sampling rate of 1 kHz.

**FIGURE 3:**
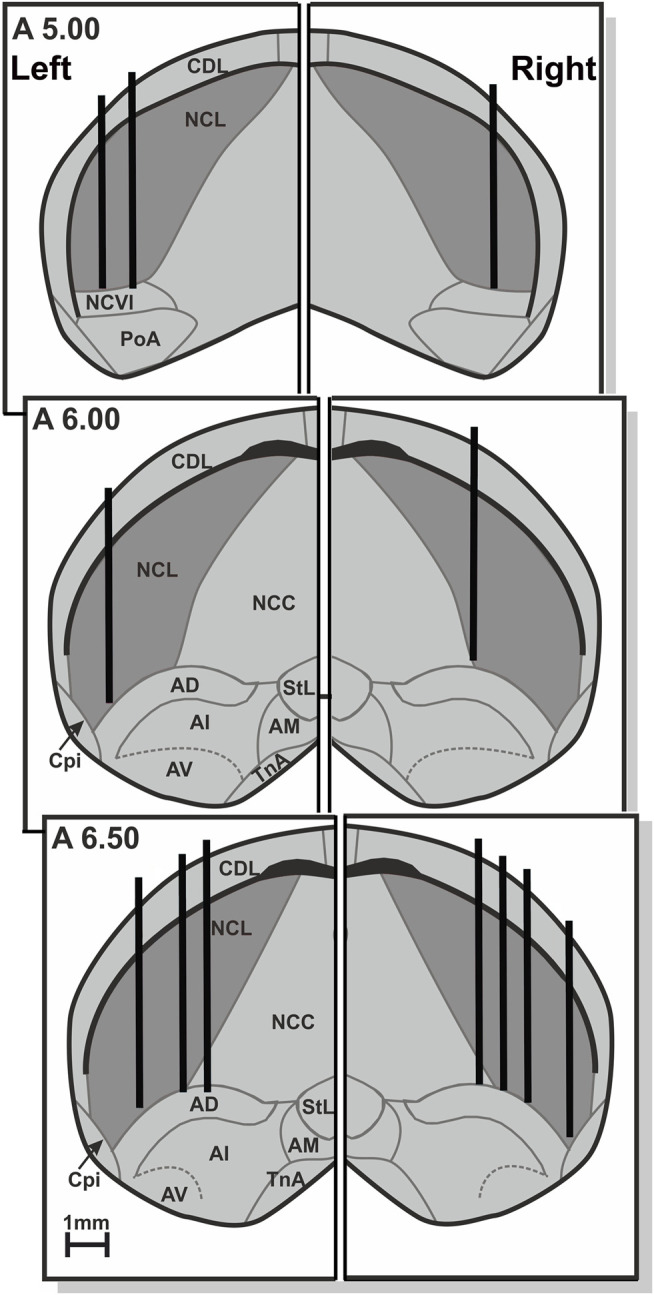
Schematic frontal depiction of the electrode positions in the left and right NCL, based on lesions and electrode tracks. Only the lateral 2/3 of the telencephalon of each hemisphere is shown, and omitting the medial 1/3. Abbreviations: AD, arcopallium dorsale; AI, arcopallium intermediale; AM, arcopallium mediale; AV, arcopallium ventral; CDL, area corticoidea dorsolateralis; Cpi, cortex piriformis; NCC, nidopallium caudale pars centralis; NCL, nidopallium caudolaterale; NCVl, nidopallium caudoventrale pars lateralis; N. PoA, posterioris amygdalopallii; StL, striatum laterale; N. TnA, taeniae amygdalae.

After experiments were finalized, birds were anesthetized with ketamine hydrochloride (initial dose of 40 mg/kg followed by supplements of 20 mg/kg/h) and xylazine hydrochloride (5 mg/kg followed by 2 mg/kg/h) into the pectoral muscle. An electrolytic lesion was placed by a 50 µA positive current (20–30 s). Then, equithesin (0.45 ml/100 g body weight) was added, the animals were perfused and brains were removed. Using the classic nissl histology, the exact positions of lesion sites and electrode tracks were determined, and were all confined in NCL (Karten and Hodos, 1967; Kröner and Güntürkün, 1999) (Figure 3).

### Data Analyses

Using a one-way analysis of variance (ANOVA), neurons were defined as task-related when their firing rates during the response or reward phase across all correct response trials were significantly different with spontaneous activities at a same length of time period during ITI before each trial. The data were quantitatively analyzed off-line by Spike2 software (CED) and custom-made MATLAB routines. Single units were classified based on full wave templates and clustered by principle component analysis and direct waveform feature measures. Only well isolated units were included in this study.

To calculate response onset times of each neuron to the stimulus onset or the animals’ first response, all spikes were trial-to-trial aligned to either stimulus onset or first mandibulation. We applied the trial-to-trial Poisson spike train analysis to calculate the response onset time of each neuron (Hanes et al., 1995). Response onset times of excited neurons were calculated as the mean time of the first burst of this neuron relative to stimulus onset across all correct response trials. The response onset times of inhibited neurons were the mean value of inhibitory onset time relative to the Go-stimulus onset across all correct response trials. The inhibitory onset time in each trial was the time at which the firing rate of this neuron was lower (*p* < 0.05) than the predicted one according to spike trains during ITI.

Responses of each NCL neuron were calculated based on their spike density function, which was determined after trial-to-trial aligning all spikes either to the stimulus onset or the first mandibulation during the 3 s response period. This spike density function of each neuron was estimated with kernel density estimations (Shimazaki and Shinomoto, 2010). After subtracting the mean value of spontaneous activity (10 s of ITI), the spike density function across all successful response trials was the temporally pure firing rate change. The peak response times and peak responses were calculated based on the spike density functions of each excited neuron. For inhibited neurons, the inhibitory duration was the time period between the inhibitory start and end time, which was the intersection of the spike density function of each neuron after stimulus onset with the mean activity minus standard deviation of spontaneous responses during ITI.

To quantify the response selectivity of each neuron to Go-/NoGo-stimuli, the spike density function of each neuron to Go- and NoGo-stimuli during 3 s of response period was compared with a receiver-operating characteristic (ROC) analysis. Each point of ROC curve depicted the proportion of bins (20 ms) on which the NoGo-responses exceeded a criterion level against the proportion of bins on which the Go-responses exceeded the same criterion. The criterion level was increased from minimum spikes per bin to the maximum one in one-spike increments. The area under the ROC curve (AUROC) gives the strength of firing that varies in a range from 0 to 1. A value of 0.5 indicates a complete overlap of neuronal responses to Go- and NoGo-stimuli, whereas a value of 0 or 1 indicates a perfect separation. If the AUROC-value of the excited neuron is closer to 1, it implies a higher selectivity of this neuron to the Go-than to the NoGo-stimulus. The AUROC-value of inhibited neuron close to 0 implies a higher discrimination ability.

## Results

### Pigeons Learn and Respond Faster to Stimuli Presented to the Right Eye

All six pigeons used in the current experiment reached the learning criterion faster when the stimuli were presented to the right eye (left-eye: 48.5 ± 14.9 sessions; right-eye: 25.7 ± 8.4 sessions; mean ± SEM; two-tailed Wilcoxon signed-rank test, *p* = 0.03). Once animals mastered the task, the subsequent single-unit recordings in NCL were started. Across all recording sessions, all animals maintained a high correct response rate to Go- and NoGo-stimuli in two monocular viewing conditions (left-eye: 86 ± 0.41%; right-eye: 85.5 ± 0.45%; n = 115 sessions). On average, pigeons responded faster when the stimuli were presented to the right eye (left-eye: 1.53 ± 0.1 s; right-eye: 1.21 ± 0.11 s; mean ± SEM; two-tailed paired *t*-test: *p* = 0.04).

### Go-Stimuli Drive NCL Visuomotor Neurons Which Then Remain Active Until the Animals’ Behavioral Onset

**FIGURE 4:**
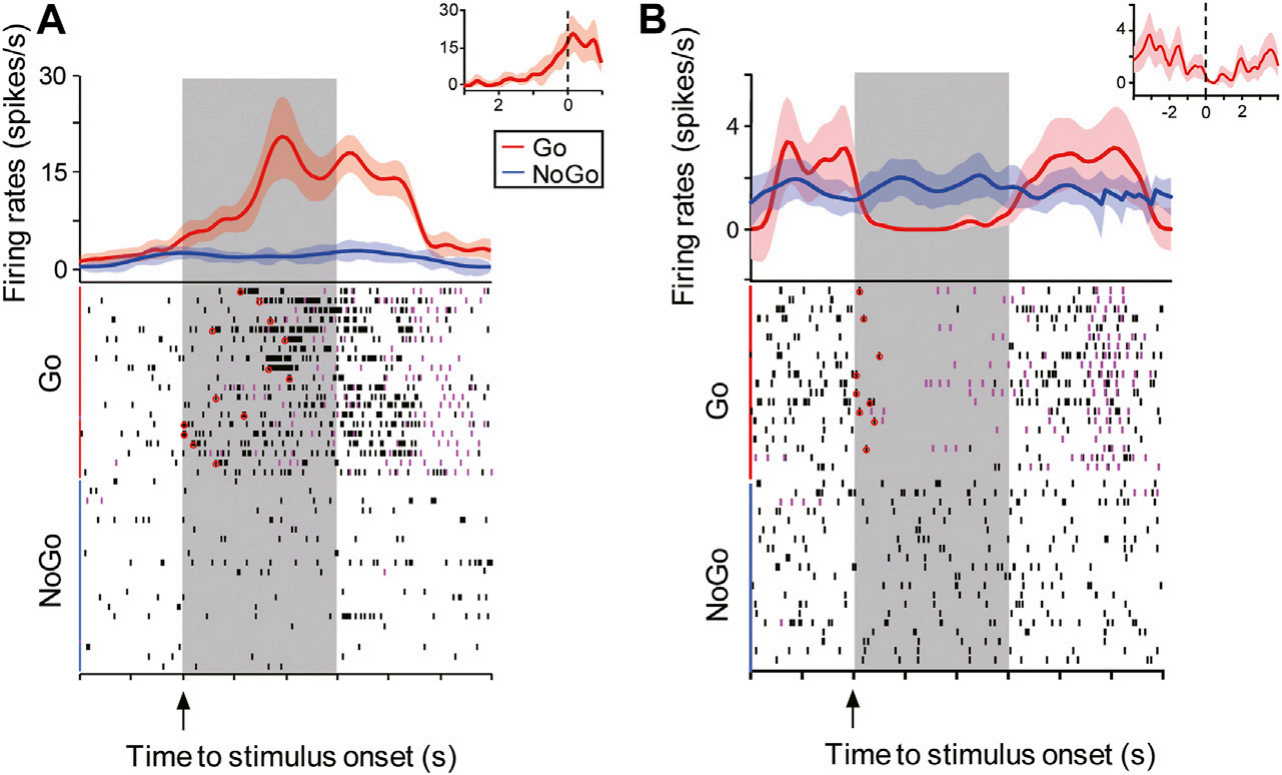
Representative example of an excited and an inhibited visuomotor neuron in the NCL. Animals had 3 s (gray shading) to either respond (magenta ticks: mandibulations) to the Go-stimulus (red line) or refrain from responding to the NoGo-stimulus (blue line). The visuomotor neurons were either excited **(A)** or inhibited **(B)** by Go-stimuli (mean ± SEM), but they did not respond to the NoGo-stimuli. The neuronal onset time for each trial was determined by Poisson spike train analyses and are marked with hollow red circles in the raster plots shown in the lower half. After trial-to-trial aligning all spikes of each neuron to the animal’s first mandibulation (zero) during the 3 s response period, these neurons showed preceding excitatory or inhibitory activity before the animal’s first response (insets, mean ± SEM)*.*

In total, we separated 627 NCL neurons in the experiment. In order to link the neuronal activities with the animals’ behavior, we trial-to-trial aligned all spikes of each neuron either to the stimulus onset or the animal’s first jaw movement (mandibulation) during the 3 s response period. We then calculated the peristimulus time histogram (PSTH) of each neuron by averaging all spikes across aligned trials. From PSTHs aligned with the stimulus onset, we found that 309 neurons (150 left, 159 right) were stimulus-driven. Each animal contributed 25 ± 9 neurons and 26 ± 7 neurons (mean ± SD, n = 6 animals) from the left and right NCL, respectively. By comparing the activity of these neurons during task execution with their spontaneous activity during ITI, we could separate neurons into two groups. They were either excited or inhibited by Go-stimuli, but were not affected by NoGo-stimuli. Depending on their response patterns to Go-stimuli, 233 neurons were classified as excitatory (113 left, 120 right) and 76 neurons as inhibitory (37 left, 39 right). Based on PSTHs aligned with the animal’s first mandibulation, some task-related neurons showed preceding responses before the animals’ first mandibulation to Go-stimuli (Figure 4). Since these neurons were driven by visual inputs and started their excitatory or inhibitory responses prior to the motor output, we dubbed these neurons excited or inhibited visuomotor neurons. These visuomotor neurons responded either to input from one eye (contralateral or ipsilateral) or from both eyes (excited neurons: 23 left, 26 right; inhibited neurons: 24 left, 26 right). Since error rates were very low, our results did not contain a separate analysis of trials with misses or false alarms.

### Left and Right Visuomotor Neurons Respond Equally Fast to Go-Stimuli

Remember that pigeons were faster to respond when seeing the Go-stimuli with the right eye (left hemisphere). In order to investigate neuronal correlates of speed differences of left-right responses, we analyzed the spike latencies to Go-stimuli. To this end, we applied trial-to-trial Poisson spike train analyses and calculated response onset times of excited (ExFSt) and inhibited visuomotor neurons (InFSt) to Go-stimulus onsets. ExFSt-values of excited neurons give the mean time of the first burst relative to Go-stimulus onset across all correct response trials. Correspondingly, InFSt-values of inhibited neurons give mean values of inhibitory onset times to Go-stimulus onsets which were calculated as the time at which firing rates started to be lower (*p* < 0.05) than the predicted one according to ITI spike trains. Since visuomotor neurons did not respond to NoGo-stimuli, such values were not available for this stimulus class.

**FIGURE 5:**
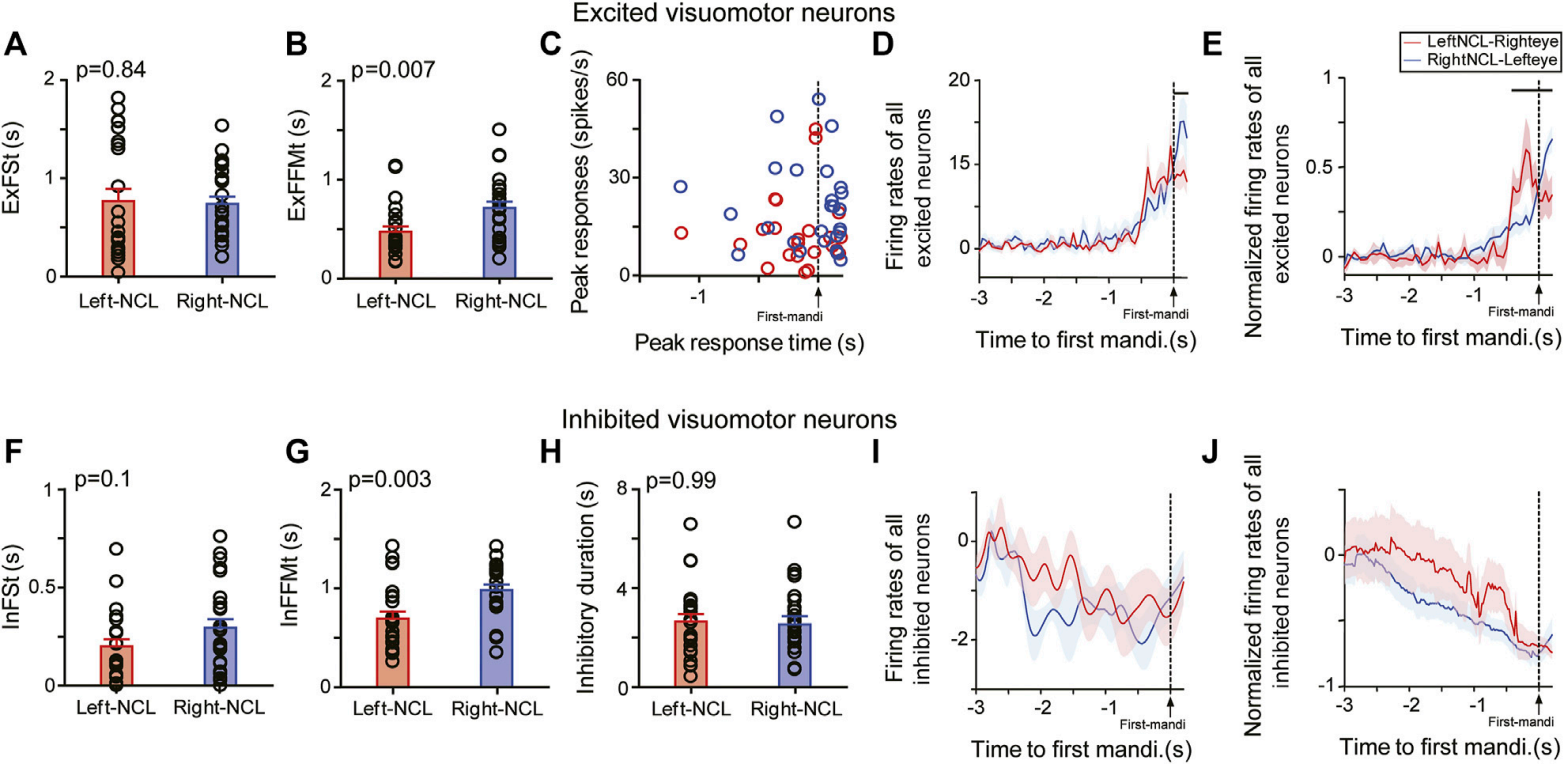
Responses of excited (upper row) and inhibited (lower row) visuomotor neurons to Go-stimuli presented to the contralateral eye. Mean response onset time (± SEM) of excited **(A)** and inhibited neurons **(F)** to Go-stimuli. Mean latency (± SEM) between neuronal and behavioral onset of excited **(B)** and inhibited neurons **(G)**. **(C)** Peak responses and peak-firing times of excited neurons. Mean firing rates (± SEM) of left and right excited **(D)** or inhibited visuomotor neurons **(I)** relative to the first mandibulation of animals. Normalized mean responses (± SEM) of all visuomotor neurons relative to the first mandibulation of animals, in which firing rates of each neuron were normalized to its peak responses of excited neurons **(E)** or lowest responses of inhibited neurons **(J)**. Horizontal lines indicate time points of significant firing rate differences between left and right NCL excited neurons (p < 0.05). **(H)** Duration of inhibitory responses of inhibited neurons.

Excited and inhibited visuomotor neurons of both hemispheres revealed no differences in response onset times to contralateral Go-stimuli. For excited neurons, ExFSt-values were 0.77 ± 0.13 s for left (mean ± SEM, n = 23) and 0.74 ± 0.07 s for right NCL neurons (n = 26) (two-tailed *t*-test: *p* = 0.84; Figure 5A). Similarly, inhibited NCL neurons of both hemispheres had comparable inhibitory onset times relative to Go-stimuli (InFSt: left-NCL: 0.2 ± 0.04 s, n = 24; right-NCL: 0.29 ± 0.04 s, n = 26; mean ± SEM, two-tailed *t*-test: *p* = 0.1; Figure 5F). Thus, neuronal spike onset times could not explain hemispheric differences in response speed of the birds. However, these analyses showed that inhibited neurons of both hemispheres responded faster to Go-stimuli than excited ones (left and right NCL: *p*’s < 0.0002; Figures 5A,F).

### The Left Hemisphere is More Efficient in Activating the Motor Output

To evaluate the latency between the neuronal response and the animal’s movement onset, we calculated the ExFFMt-value for excited and InFFMt for inhibited neurons. The ExFFMt-value of each excited neuron is the mean latency between the first burst and the animals’ first mandibulation across all correct response trials. Similarly, InFFMt-values are the latencies between inhibitory onset and the animals’ first mandibulation across all correct response trials.

Our results showed that after Go-stimulus onset, the excited visuomotor neurons in left NCL had shorter ExFFMt-values than those in right NCL (left: 0.47 ± 0.05 s, n = 23; right: 0.71 ± 0.06 s, n = 26; mean ± SEM *p* = 0.007) (Figure 5B). Thus, NCL neurons of the left hemisphere were faster in turning their activation into an appropriate motor output. But which mechanism produced this left-right difference? To find an answer, we looked at the neuronal dynamics of NCL neurons. First, we compared Go-stimulus driven peak-firing times of excited neurons. As shown in Figure 5C, the majority of left NCL neurons reached their peak activities before the animals’ first mandibulation (left: 74%, 17/23; right: 35%, 9/26; two-tailed chi-square test: *p* < 0.01). To analyze the relation between the timing of the motor output and the neuronal activity of NCL neurons, we compared the activities of these neurons 200 ms before and during the animal’s first mandibulation. The mean firing rates of left NCL neurons were comparable shortly before and after the behavioral motor output (before: 6.55 ± 1.48 spikes/s; during: 4.48 ± 1.49 spikes/s; *p* = 0.22). On the contrary, the neuronal activities of right NCL neurons continuously increased after the onset of the animal’s first mandibulation (before: 9.17 ± 1.93 spikes/s; during: 16.66 ± 2.85 spikes/s; *p* < 0.0003). No inter-hemispheric activity differences were observed before the animal’s first mandibulation (*p* = 0.35). However, right NCL neurons reached higher peak firing rates than those on the left but this peak came after mandibulation (*p* = 0.004) (Figure 5D). We then normalized the firing rates of each neuron to the peak value during 3 s before the animal’s response. As shown in Figure 5E, normalized firing rates sharply increased in left NCL before the animals’ first mandibulation, while those in right NCL did so after first mandibulation. The left and right NCL neurons were significantly more active before and after the animals’ response onset, respectively (each *p* < 0.05). Thus, the left NCL was able to determine the animals’ responses by its early rise in activity. As a result, the right NCL reached its peak activity when the animals’ response had already started and consequently came too late.

We then turned our attention to the NCL neurons that were inhibited by Go-stimulus onset. These neurons displayed higher spontaneous activities during ITI than the excited ones (both left and right NCL, *p*’s = 0.03). After the appearance of Go-stimulus, inhibited neurons of the left NCL had shorter InFMMt-values than those in the right NCL (InFMMt: left: 0.69 ± 0.07 s, n = 24; right: 0.98 ± 0.06 s, n = 26; mean ± SEM, two-tailed *t*-test: *p* = 0.003) (Figure 5G). The durations of inhibitory activities were comparable between left and right NCL neurons (left: 2.65 ± 0.3 s, right: 2.64 ± 0.27 s, *p* = 0.99; Figure 5H). In each hemisphere, the activities of inhibited neurons declined slowly after Go-stimulus onset and reached its lowest level before the animals’ first mandibulation. The mean activities before and during the animal’s first mandibulation (200 ms) were comparable for neurons in the left (before: 1.27 ± 0.58 spikes/s; during: 0.99 ± 0.71 spikes/s; *p* = 0.19) and right NCL (before: 1.58 ± 0.52 spikes/s; during: 1.26 ± 0.43 spikes/s; *p* = 0.59). No significant inter-hemispheric activity differences were found before (*p* = 0.73) and during the first response of animals (*p* = 0.73) (Figure 5I). This result was also valid for normalized data patterns (Figure 5J).

### Integrating Inputs From Both Eyes

The optic nerves of birds with their laterally placed eyes cross virtually completely in the optic chiasm. Thus, the optic fibers of each eye project nearly only to the contralateral half brain (Güntürkün and Karten, 1991). At the subpallial level, the re-crossing of visual information is enabled by mesencephalic and meso-diencephalic commissural projections (Stacho et al., 2016). As shown in Figure 1, the tectorotundal commissural crossover is asymmetrically organized such that the left hemisphere integrates visual inputs from two hemispheres to a larger extent than the right hemisphere (Güntürkün et al., 1998; Letzner et al., 2020; Rogers, 2020). As a result, the left visual entopallium receives more information from both visual fields (Verhaal et al., 2012). Consequently, the projections from the entopallial system to the arcopallium create similar asymmetries of visual representation in the arcopallium (Xiao and Güntürkün, 2018). These are partly re-balanced by the exchange of left and right arcopallia through the commissura anterior (Xiao and Güntürkün, 2021).

Since NCL receives inputs from both the entopallial system as well as from the arcopallium (Kröner and Güntürkün, 1999), we expected some left-right differences in the bilateral visual integration between two hemispheres. To test this hypothesis, we used receiver-operating characteristic (ROC) analyses to compare the discrimination ability of each visuomotor neuron to Go-/NoGo-stimuli from the ipsilateral or the contralateral eye. To reiterate, an AUROC-value of 0.5 indicates that the neuron does not differentiate between Go- and NoGo-stimuli, whereas values of 0 or 1 indicate perfect separation. If the AUROC-value of an excited neuron is close to 1, it implies a higher selectivity of this neuron to the Go-than to the NoGo-stimulus. An AUROC-value of 0 indicates a perfect discrimination of an inhibited neuron.

**FIGURE 6:**
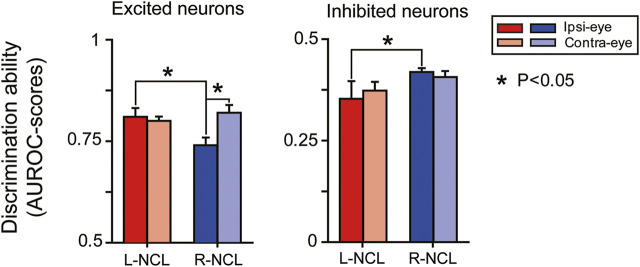
Discrimination scores (AUROC-value: mean ± SEM) of NCL visuomotor neurons in left (L) or right NCL (R)*.*

For contralateral inputs, the excited visuomotor NCL neurons did not show inter-hemispheric differences in AUROC-values (left: 0.8 ± 0.01, n = 23; right: 0.82 ± 0.02, n = 26; two-tailed *t*-test: *p* = 0.58). With respect to ipsilateral inputs, left NCL neurons reached higher AUROC-scores than those on the right (left: 0.81 ± 0.02, n = 11; right: 0.74 ± 0.02, n = 10; *p* = 0.02). Overall, left excited neurons showed comparable discrimination performances for contralateral and ipsilateral inputs (*p* = 0.45). On the contrary, right sided excited neurons evinced higher AUROC-scores for contralateral than ipsilateral inputs (*p* = 0.01; Figure 6). This was to be expected based on the more pronounced bilateral input of the left hemisphere (Figure 1).

For inhibited visuomotor neurons, no inter-hemispheric differences were observed in their AUROC-scores for contralateral inputs (left: 0.37 ± 0.02, n = 24; right: 0.41 ± 0.01, n = 26; two-tailed *t*-test: *p* = 0.58). Only for ipsilateral input, we observed superior discrimination performances of left NCL inhibited neurons compared with the right ones (left: 0.35 ± 0.04, n = 10; right: 0.42 ± 0.01, n = 20; *p* = 0.04). NCL inhibited neurons of each hemisphere showed comparable discrimination abilities for input from each eye (left: *p* = 0.65; right: *p* = 0.5; Figure 6).

**FIGURE 7:**
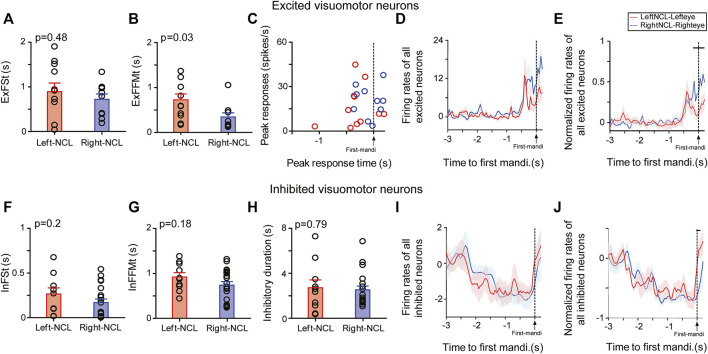
Responses of visuomotor neurons to Go-stimuli presented to the ipsilateral eye. Response characteristics of excited **(A–E)** and inhibited neurons **(F–J)** in the NCL to stimuli presented to the ipsilateral eye (ExFSt; InFSt) and their subsequent ability to swiftly activate a motor response (ExFFMt; InFFMt). Mean response onset time (± SEM) of excited **(A)** and inhibited neurons **(F)** to Go-stimuli. Mean latency (± SEM) between neuronal and behavioral onset in excited **(B)** and inhibited neurons **(G)**. **(C)** Peak activities and peak-firing times of excited neurons in left and right NCL. Mean firing rates (± SEM) of excited **(D)** and inhibited visuomotor neurons **(I)** relative to the first response of the animal to ipsilateral stimuli. Normalized mean responses (± SEM) of excited **(E)** and inhibited visuomotor neurons **(J)** relative to the first response of the animal. Firing rates of each neuron were normalized to its peak (excited neuron) or lowest response (inhibited neuron). The horizontal lines in E and J indicate the significance level of the comparison of left and right NCL neurons (p < 0.05). **(H)** Duration of inhibitory responses of inhibited neurons.

We then turned our attention to possible hemispheric speed differences in the integration of ipsilateral and contralateral input. Both excited (ExFSt: left: 0.89 ± 0.19 s, n = 11; right: 0.72 ± 0.12 s, n = 10; mean ± SEM, two-tailed *t*-test: *p* = 0.48; Figure 7A) and inhibited visuomotor neurons (InFSt: left: 0.27 ± 0.07s, n = 10; right: 0.17 ± 0.04 s, n = 20; *p* = 0.2; Figure 7F) of both hemispheres responded equally fast to Go-stimuli presented to the ipsilateral eye. Overall, inhibited neurons started their responses earlier than excited neurons (all *p*’s < 0.03).

Up to now we presented data on stimulus-driven neuronal responses. We now concentrated on whether there was a potential correlation between the response speed of NCL neurons and animals’ behavioral responses. When the ipsilateral eye was stimulated, the ExFFMt-values of excited visuomotor neurons in the right NCL were smaller than those in the left (left: 0.72 ± 0.13 s, n = 11; right: 0.34 ± 0.09 s, n = 10; mean ± SEM. two-tailed *t*-test: *p* = 0.03; Figure 7B). Thus, right NCL was faster in translating an ipsilateral Go-stimulus input into an appropriate motor response.

The excited neurons in both hemispheres did not differ much in their general response properties to ipsilaterally presented stimuli. They had comparable peak activities (left: 16.5 ± 4.2 spikes/s; right: 20 ± 3.4 spikes/s; *p* = 0.53; Figure 7C), evinced comparable peak-firing times (left-NCL: 0.31 ± 0.1 s; right-NCL: 0.07 ± 0.07 s; *p* = 0.07; Figure 7C), and showed no hemispheric differences before (*p* = 0.08) and during the animal’s first response (*p* = 0.09; Figure 7D). However, after firing rates of excited neurons were normalized relative to their peak responses, we found that more left than right NCL neurons reached their peak responses before the animal’s first mandibulation (left: 82%, 9/11; right: 60%, 6/10; Figures 7C,E).

The inhibited visuomotor neurons had higher spontaneous activities during ITI than excited ones (both left and right NCL, *p*’s = 0.04). Inhibited neurons had comparable InFFMt scores between both hemispheres (left: 0.92 ± 0.1 s, n = 10; right: 0.74 ± 0.08 s, n = 20; mean ± SEM, two-tailed *t*-test: *p* = 0.18) as well as in their durations of inhibitory responses (left: 2.71 ± 0.69 s, right-NCL: 2.53 ± 0.3 s, *p* = 0.79; Figures 7G,H). After the animal’s first response, the effect of inhibition significantly similarly increased over time for both left and right inhibited neurons (both *p*’s < 0.05; Figure 7I), but evinced a rebound during the response (Figure 7J).

In summary, both excited and inhibited visuomotor neurons evinced comparable discrimination performances between two hemispheres for visual input from the contralateral eye. An asymmetry emerged, however, when visual stimuli were given to the eye ipsilateral to the recorded hemisphere. Here, left sided neurons had an advantage. In addition, the right hemisphere was more efficient in activating a motor output after ipsilateral right-eye input.

## Discussion

In the current study, we conducted single unit recordings from the “prefrontal” NCL while pigeons were working on a color discrimination task. As expected, our birds learned the discrimination faster and also responded more quickly to the Go-stimuli when using the right eye. Our recordings uncovered two main asymmetrical neuronal properties of the NCL that possibly constitute parts of the neuronal foundations of this lateralized behavior. First, both left and right NCL visuomotor neurons responded equally fast to the Go-stimuli in this study, while the response properties of left hemispheric neurons suggested that they possibly play a more important role in controlling the operant response than right NCL neurons. Thus, behavioral asymmetry of response speed does not seem to be related to encoding stimuli but to action systems. Second, the processing of stimuli from the ipsilateral eye differed between two hemispheres, with the left NCL being more efficient in representing inputs from both eyes. We will discuss these points one by one and will at the end combine them with insights from previous publications (Verhaal et al., 2012; Güntürkün et al., 2018; Xiao and Güntürkün, 2018; Xiao and Güntürkün, 2021) to propose a model of lateralized color discrimination learning and task execution in birds.

### Behavioral Asymmetries

Most studies on visual discrimination of various visual features in birds revealed faster learning and response speeds as well as higher discrimination scores when using the right eye/left hemisphere (Galliformes, domestic chicken: Vallortigara, 1989; Regolin et al., 2003; Rogers 2014; quail: Valenti et al., 2003; Gülbetekin et al., 2009; Columbiformes, pigeon: Güntürkün, 1985; Prior et al., 2004; Passeriformes, zebra finch: Alonso, 1998). Thus, most but not all avian species (Templeton and Gonzalez, 2004) evince a left hemispheric superiority in learning and discriminating visual features during appetitive tasks. Our results accord with this general pattern. It is important to mention that these findings do not signify an overall left hemispheric “visual dominance” but hint to hemisphere specific differences in the processing of visual object features. Visually guided social (Rosa-Salva et al., 2012) and spatial tasks (Tommasi et al., 2012) often reveal an inverted asymmetry pattern (Güntürkün, 1997b; Vallortigara, 2000; Regolin et al., 2005). This implies that the lateralized neural processes that we describe are related to our task contingencies and are thus amenable to change when pigeons work on a different kind of visual discrimination.

### The Left Hemisphere’s Advantage in Activating the Response

Visuomotor neurons of both left and right NCL responded with equal speed to the onset of the Go-stimuli. Thus, we did not reveal any asymmetries of stimulus encoding in the NCL. However, we observed asymmetries of response initiation. Normalized firing rates of the left visuomotor neurons increased shortly *before* the animals’ response onset while those on the right increased their spike frequencies *after* the onset of the behavioral response. Thus, if both hemispheres would rush to ignite motor areas in order to activate the behavioral response, the right NCL would mostly be too late to determine the action of the animal. This finding has two implications. First, the time advantage of the left hemisphere is, at least with respect to the NCL, not due to left-right differences of stimulus encoding but due to asymmetries of motor activation. Second, our results from the NCL are practically identical to findings from the arcopallium (Xiao and Güntürkün, 2018) and so possibly result from a similar mechanism.

As depicted in Figure 1, the arcopallium projects to subpallial motor areas and has commissural projections through the commissura anterior to the contralateral arcopallium. Xiao and Güntürkün (2018) observed that blocking neural activities of the leading left arcopallium drastically increased the variability of the ExFSt and ExFFMt values of right arcopallial neurons, but blocking right arcopallium had no such effects on left NCL neurons. This implies that the left (but not the right) arcopallium was able to control the temporal activity structure of contralateral arcopallial neurons. As a result, the left arcopallium was able to delay peak activity times of right arcopallial neurons, thereby monopolizing the control of the animals’ behavior during color discrimination. This mechanism depended on the arcopallial interactions through the commissura anterior. The NCL has no commissural projections to the other hemisphere and thus cannot invoke a similar mechanism to its homotopic side (Letzner et al., 2016). This implies that the asymmetries of the spike peak times of left and right NCL neurons possibly result from projections of the arcopallium onto the ipsilateral NCL (Figure 1).

A comparison of the current study on the NCL with those on the arcopallium (Xiao and Güntürkün, 2018) reveals an important difference in the proportion of excited visuomotor neurons. In the NCL (current study), 49% of left and 50% of right hemispheric neurons were excited by the Go-stimuli. In the arcopallium, however, the comparable numbers were 67% on the left and 38% on the right (Xiao and Güntürkün, 2018). Since the experimental procedures were identical in the current and the previous study, it is likely that the proportion of excited visuomotor neurons is indeed symmetric in NCL but asymmetric in the motor arcopallium. The small proportion of excited right hemispheric arcopallial neurons could constitute a further mechanism with which behavioral responses during visual discrimination are primarily controlled by the left hemisphere.

### Asymmetries of Visual Integration

As depicted in Figure 1, the left hemispheric entopallium receives a strong input from both the left and the right tectum through the n. rotundus (Güntürkün et al., 1998; Letzner et al., 2020). This is different from the right entopallium that mostly receives input from the contralateral eye *via* the right tectorotundal system. This anatomical asymmetry results in a higher level of bilateral representation in the left hemispheric tectofugal system (Letzner et al., 2020). The left-right difference of visual representation is also visible at the neuronal processing level (Verhaal et al., 2012; Xiao and Güntürkün, 2018) and concomitantly affects behavioral performances (Güntürkün and Hahmann, 1999; Valencia-Alfonso et al., 2009). Since the tectofugal system feeds into the NCL, we could also demonstrate an asymmetry of visual representation when comparing the input from the ipsilateral eye between left and right NCL. While AUROC values from the contra- and the ipsilateral eye did not differ in the left NCL, those in the right NCL were worse for input from the ipsilateral eye. Thus, asymmetries of bilateral visual representation of left and right NCL resemble those of the entopallium.

Xiao and Güntürkün (2021) observed a different pattern in the arcopallium. Here, the commissura anterior was the gateway to exchange visual stimuli in a reversed asymmetrical manner such that the left arcopallium provided the right side with more information about its ipsilateral visual half-field than vice versa. Thus, the arcopallial interhemispheric communication acted in an opposite direction to asymmetries of the tectofugal system, thereby leveling left-right differences of visual representation at arcopallial level. The fact that we did not observe anything comparable in the NCL implies that this symmetrizing of visual representation is confined to the arcopallium and is not projected back onto the NCL.

### A Working Hypothesis for Lateralized Discrimination Learning and Task Execution at Pallial Level in Birds

Based on the results of the present study as well as previous publications we will now outline a hypothesis on the neuronal processes during visual feature discrimination in pigeons (Verhaal et al., 2012; Güntürkün et al., 2018; Xiao and Güntürkün, 2018; Xiao and Güntürkün, 2021). We will proceed in four steps that are related to the numbers depicted in Figure 1.

First, the tectofugal system of the left hemisphere shows a higher propensity to spontaneously respond to color stimuli - an asymmetry that is drastically increased once a color is associated with reward (Verhaal et al., 2012). This is especially visible for the initial phasic burst of entopallial activity (Colombo et al., 2001) that is only visible in the left entopallium (Verhaal et al., 2012). Learning to associate a visual feature with a strong appetitive value quickly recruits a large number of left entopallial neurons that start to respond to the Go-stimuli. This left-skewed quantitative asymmetry in neuronal population size could give the left tectofugal system an important advantage to ignite neuronal networks in downstream associative and motor areas.

Second, entopallial neurons respond to a large number of visual features (Scarf et al., 2016; Clark and Colombo, 2020; Clark et al., 2022) that are represented as population codes (Koenen et al., 2016; Azizi et al., 2019). If some of these features become associated with reward, left entopallial neurons excel in discriminating between rewarded and non-rewarded stimuli and this left hemispheric superiority correlates with the lateralized responses of the animals when working under monocular conditions (Verhaal et al., 2012). Thus, visual processes and response patterns become linked.

Third, the previous point implies that activity patterns of the left tectofugal system gain a higher prediction for the upcoming reward during choice situations. Since the increase of associative strength between a visual feature and reward is proportional to the magnitude of error between prediction and outcome, we would expect a higher gradient descent of error for visuo-associative and visuo-motor networks in the left hemisphere (Rescorla and Wagner, 1972; Kruschke, 1992; Soto and Wasserman, 2010). NCL, arcopallium and striatum are densely innervated by dopaminergic fibers from the brainstem (Wynne and Güntürkün, 1995; von Eugen et al., 2020) that activate local D1A- and D1D-receptors (Herold et al., 2011; 2018) which mediate synaptic stimulus-response associations in birds (Izawa et al., 2001; Yanigahara et al., 2001; Herold et al., 2012). Thus, the capacity of the left entopallium and its associated nidopallial and mesopallial territories (Stacho et al., 2020) to better discern between the rewarded and non-rewarded features would result in a dopamine-mediated higher synaptic coupling between the visual tectofugal, “prefrontal” and arcopallial motor neurons in the left hemisphere (Güntürkün et al., 2018).

Fourth, the commissura anterior plays a key role in the lateralized transduction of these visual and associative processes into the animal’s response pattern. Xiao and Güntürkün (2018) could show that the left arcopallium can adjust the responses of right arcopallial neurons such that their motor command is generated too late to influence the choice of the pigeon. As a consequence, the left hemisphere dominates choices and responses of the animal. A further function of the commissura anterior is to re-balance the asymmetries of bilateral visual representation between two hemispheres such that left and right arcopallia have equal access to ipsi- and contralateral information during motor execution (Xiao and Güntürkün, 2021). Thus, different functions of which some increase and others decrease functional asymmetries are accomplished *via* the commissura anterior.

Our working hypothesis covers the loop that starts with the midbrain tectum, proceeds to the thalamic n. rotundus and the pallial entopallium and runs up to the “prefrontal” NCL and the motor arcopallium. Our ideas are obviously speculative at the moment but can serve as a working hypothesis for future experiments. These could, for example, use a meta-conflict paradigm, where both hemispheres are occasionally brought into a response conflict (Adam and Güntürkün, 2009; Ünver and Güntürkün, 2014; Manns et al., 2021). Such conflicts result in slower response times and an outcome in which the perceptual specialization of one hemisphere often dominates that of the other. In this case, we would expect the left hemisphere to more often dominate the animals’ behavior. The situation is different, when both eyes synergistically see their specific Go-stimulus. This condition was dubbed “super-stimulus” in the study of Ünver and Güntürkün (2014) and resulted in faster response times. All of these studies were purely behavioral and thus it is speculative what the neuronal response patterns would look like. But such a design is appropriate to test our hypothesis of a commissural mechanism that swiftly enables a shift between interhemispheric competition and cooperation.

Overall, this scenario uncovers many open questions that remain to be answered in order to come up with fully mechanistic explanations of the processes that govern avian visual asymmetries. Foremost is the question why left tectofugal neurons are more apt to respond to neutral visual cues and are subsequently superior in discriminating between visual features, once discrimination training has started. Answers to these and similar questions are needed to achieve a truly comprehensive account that goes beyond the preliminary hypothesis that the current study can provide.

## Data Availability

The raw data supporting the conclusions of this article will be made available by the authors, without undue reservation.
